# Supplementary material for: Improving detection of protein-ligand binding sites with 3D segmentation
Source: Sci Rep. 2020 Mar 19;10:5035. doi: 10.1038/s41598-020-61860-z (PMC7081267; doi:10.1038/s41598-020-61860-z)
Supplement: Supplementary file 1 — Supplementary Information. [file 41598_2020_61860_MOESM1_ESM.pdf]

Improving detection of protein-ligand binding sites with 3D segmentation.

Marta M. Stepniewska-Dziubinska[1], Piotr Zielenkiewicz[1,2], and Pawel Siedlecki[1,2,*]

[1]Institute of Biochemistry and Biophysics, Polish Academy of Sciences, Pawinskiego 5a, 02-106 Warsaw, Poland
[2]Department of Systems Biology, Institute of Experimental Plant Biology and Biotechnology, University of Warsaw, Miecznikowa 1, 02-096 Warsaw, Poland

[*]pawel@ibb.waw.pl

Supplementary Information

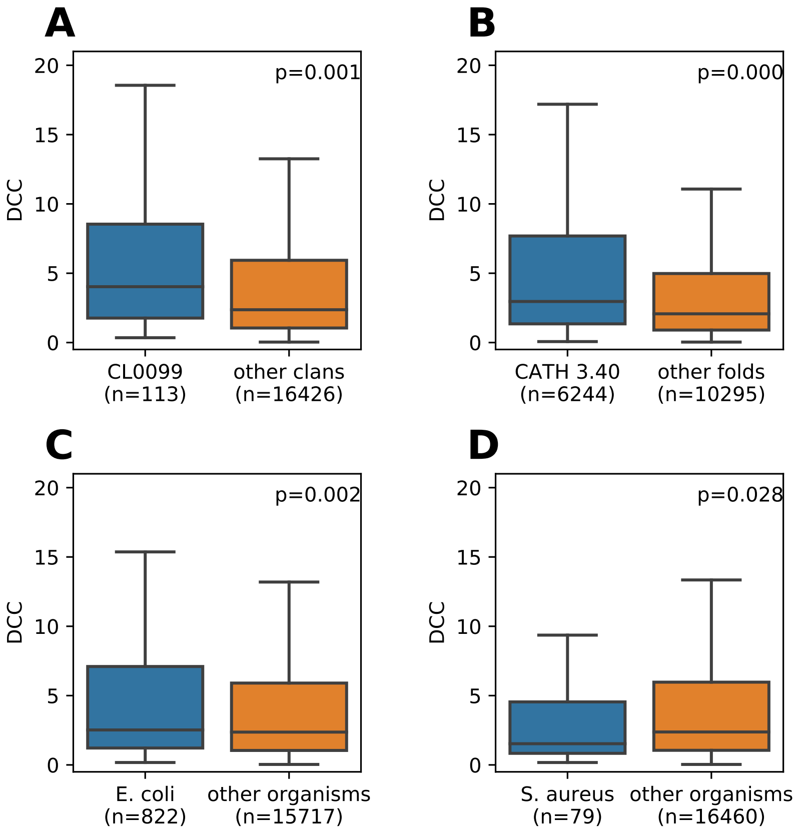

Supplementary Figure S1. Relationship between prediction accuracy and protein properties: superfamily (A), fold (B), and source organism (C and D). "n" denotes number of binding sites in each group. p-values were calculated with Mann-Whitney U-test.
